# The Bright, Artificial Intelligence-Augmented Future of Neuroimaging Reading

**DOI:** 10.3389/fneur.2017.00489

**Published:** 2017-09-21

**Authors:** Nicolin Hainc, Christian Federau, Bram Stieltjes, Maria Blatow, Andrea Bink, Christoph Stippich

**Affiliations:** ^1^Division of Diagnostic and Interventional Neuroradiology, University of Basel, University Hospital Basel, Basel, Switzerland; ^2^Clinic for Radiology and Nuclear Medicine, University of Basel, University Hospital Basel, Basel, Switzerland

**Keywords:** neuroradiology, radiology, artificial intelligence, machine learning, neuroimaging, magnetic resonance imaging

## Abstract

Radiologists are among the first physicians to be directly affected by advances in computer technology. Computers are already capable of analyzing medical imaging data, and with decades worth of digital information available for training, will an artificial intelligence (AI) one day signal the end of the human radiologist? With the ever increasing work load combined with the looming doctor shortage, radiologists will be pushed far beyond their current estimated 3 s allotted time-of-analysis per image; an AI with super-human capabilities might seem like a logical replacement. We feel, however, that AI will lead to an augmentation rather than a replacement of the radiologist. The AI will be relied upon to handle the tedious, time-consuming tasks of detecting and segmenting outliers while possibly generating new, unanticipated results that can then be used as sources of medical discovery. This will affect not only radiologists but all physicians and also researchers dealing with medical imaging. Therefore, we must embrace future technology and collaborate interdisciplinary to spearhead the next revolution in medicine.

## We have an Intrinsic Fear of Accepting New Technology

To help better understand the brain pathologies of patients, radiologists provide qualitative and quantitative exam results based on well-established methods in cross-sectional neuroimaging such as magnetic resonance imaging (MRI) and computed tomography (CT); ultimately, we must integrate new advancements in computer technology to further evolve patient care as summarized by Kassubek (1). This step may seem logical, yet the introduction of the first hand-held scientific calculator in 1972 was met with apprehension; engineers would supposedly lose their basic tools of the trade (2). Similarly, the dawning age of artificial intelligence (AI) has left experts discussing the future existence of human radiologists, questioning their necessity after AI has been fully established. The concern seems valid to a degree—humans cannot match the work rate and accuracy of machines, qualities which become increasingly valuable as radiological exam loads increase (3) and a doctor shortage looms (4). As if, with the arrival of AI-based automation, radiologists will be reduced to the upkeep and maintenance of these superior beings while exam results are sent directly to clinicians. The more likely scenario, however, is quite the opposite. We expect that the development of AI will lead to complementary, statistical information available from medical imaging, effectively creating an “augmented radiologist.” Engineers were liberated from the tedious task of calculation and could concentrate on innovation, essentially jump-starting the surge in computer technology. Radiology may experience a renaissance—and a progression in medicine will ensue—but only with the help of clinicians.

## Silently, The Foundation for the Era of AI in Radiology was Laid Decades Ago

The ability to store medical image information through the introduction of a digital picture archiving and communication system (PACS) was advantageous for many reasons—film transport, processing, filing, and retrieval was no longer necessary as images could be read on- and off-site instantaneously, workflow was optimized, and communication between physicians was improved. As a result, decades’ worth of readily available pathologic and anatomic patient scan information now lies in hospital servers waiting to serve a higher purpose; robust and efficient big data solutions present the only considerable hurdle to unlocking potential medical breakthroughs. An example of a radiological big data solution, known as PACS mining, was presented by Re et al. at ESR Vienna 2017 (5), where an algorithm designed to automatically extract bone data from computed tomography (CT) scans in the PACS found an inverse relationship of bone density to increasing age. While the result is no surprise, it is an example of how patient data from one million CT samples can be post-processed by a single user in a clinically feasible timeframe.

Mining the PACS for MRI data, however, provides additional challenges. The heterogeneous collection of images due to differing pulse sequences and sequence parameters within an institution, let alone across multiple centers, provide a hurdle to big data analysis. Ideally, well-organized databases using standardized protocols are constructed to minimize variation across the data to produce viable results. In Switzerland, the Swiss Multiple Sclerosis Cohort study is at the forefront of this reform, standardizing MRI protocols across seven Swiss multiple sclerosis centers (6). This cohort can then be used in big data analysis and provide a stepping stone toward the implementation of AI—the homogeneous dataset can be used to train the AI before it is applied to its defining task of recognizing patterns in more heterogeneous data. Big data solutions for MRI are currently being sought in projects such as the human connectome project (7–9), the BRAIN initiative (10), and the Organization of Human Brain Mapping (11) have launched various initiatives seeking to deepen our understanding of healthy brain function, neurological and psychiatric disorders through the mapping of brain areas and connections.

Artificial intelligence is defined as “the theory and development of computer systems able to perform tasks normally requiring human intelligence, such as visual perception … and decision-making”^1^ and has already been shown to work in image-based diagnostics. A pathology research group at Stanford found that AI was able to accurately differentiate between two different forms of lung cancer on histopathology images, predicting survival rates better than pathologists (12). Radiology, like pathology, is image based; input data from images can be provided to algorithms, where characteristic image features are extracted and clustered into similar classes. Due to the vast amount of data in pathology, data sampling is already the rule, not the exception (13), as analyzing the entire tissue specimen is not feasible in a clinical timeframe. Radiology does not rely on sampling, but with an estimated allotted time of just 3 s per image to meet current workload demands (3), it is no secret that radiologists could use some help. Automated detection algorithms based on AI could reduce the workload; benefits of AI in radiology have already been shown in the identification and classification of microcalcification in mammography (14) and has been tested against human second readings for the purposes of breast cancer screening (15).

## Clinicians Hold the Key to Launching an Innovative AI in Radiology

An easily searchable, fully digital clinical information system including access to digitally stored exam results could allow the radiological AI to correlate images with patients’ longitudinal outcome (e.g., treatment response and survival rates), allowing for the extraction of new imaging biomarkers driven by prognostic information. One such example is texture analysis of tumors. Many papers are currently geared toward finding differences in tumor textures and correlating this to histopathological and molecular data in an attempt to extract prognostic information from medical imaging alone. In order for an AI to do this, not only are robust fully automated 3D volumetric segmentation algorithms required (16), the tissue characterization data must be readily available in digital form for correlation. The AI can then use treatment data and survival rates and directly correlate this to initial texture information; the next time the AI finds a certain texture, it may indicate that the patient requires a more aggressive form of therapy to begin with.

Clinicians have the unique opportunity to motivate patients to employ wearables or even make use of their mobile phones to collect data for longitudinal follow up; essential data to the AI in cases where the patient does not return to the hospital. Accelerometers in mobile phones can already be used to quantify movements in Parkinson’s disease (17), which could be used by the radiological AI looking to interpolate data back before the patients’ initial MRI using statistical image analysis software (18, 19) to extract biomarkers visible before disease onset. Effectively, longitudinal clinical information supplied by clinicians will result in an AI with super-human abilities.

## Is There Anything Stopping AI from Completely Replacing the Radiologist?

Artificial intelligence must be contrasted to general intelligence, an essentially human trait. For example, a radiologist will intuitively recognize a swallowed foreign body on radiograph as a coin, while the AI will merely detect an anomaly. Moreover, while an AI, based on the training and analysis of super-human amounts of medical images might one day characterize lesions as good as (or better than?) a physician and suggest new subcategories of disease, it will be the task of the radiologist to interpret the findings in a global understanding of facts which will be intrinsically interdisciplinary. Accurately diagnosing a new disease by AI also takes time as it requires millions of data sets to reach an acceptable level of accuracy and biases in data could lead to skewed results (20).

Second, informed consent and transparent decision-making are at the foundation of patient care. Yet, the manner in which AI derives results is often unclear—the networks are free to organize themselves, giving rise to an appropriately termed black box analogy. For example, on February 15, 2011, IBM’s supercomputer Watson famously answered “Toronto” to a question in the final round of jeopardy regarding airports in U.S. cities. More important than the wrong answer though, was the fact that the computer’s train of thought could not be elucidated by experts. The black box of AI might fail in ways we do not expect; humans must act as a safeguard for the erroneous results in an essential AI process known as keeping the “human in the loop.”

## Adapting to AI Will be a Challenge…

…yet yield great rewards. AI will inevitably produce unanticipated results that are not as obvious as the Toronto outlier above. Physicians will need to adapt to deal with these results—at what point do we stop disregarding these seeming “outliers” (or potentially new subcategories of disease) and start using them as possible sources of medical discovery? As additional information is derived from the use of statistical analysis of medical imaging data, combined with longitudinal follow-up information supplied by clinicians, our understanding of disease will be challenged with definite implications in treatment and the improvement of patient care. The new subcategories of disease created by AI can be directly applied to precision medicine (21), which seeks to provide targeted therapy based on a patient’s individual lifestyle, environment, and biology under the motto “the right treatment for the right patient at the right time.” Let us embrace future technology and spearhead the next revolution in medicine together.

## Author Contributions

All authors listed have made a substantial, direct, and intellectual contribution to the work and approved it for publication. NH: research, manuscript writing, and editing. CF, BS, MB, AB, and CS: research, manuscript writing, and revision.

## Conflict of Interest Statement

The authors declare that the research was conducted in the absence of any commercial or financial relationships that could be construed as a potential conflict of interest.

## Footnotes

^1^ en.oxforddictionaries.com.
